# Editorial: Insights in health informatics-2021

**DOI:** 10.3389/fdgth.2022.1129054

**Published:** 2023-01-09

**Authors:** Daihai He, Salihu S. Musa

**Affiliations:** Department of Applied Mathematics, Hong Kong Polytechnic University, Hong Kong SAR, China

**Keywords:** digital health, healthcare, effectiveness, smart devices, health conditions

**Editorial on the Research Topic** Insights in health informatics-2021

The globe is constantly evolving due to technological development. Digital health incorporates digital systems and technology with health, healthcare, and society to improve the effectiveness of healthcare delivery and healthcare practice (1, 2). It aids healthcare professionals in responding to patients’ needs more efficiently and cost-effectively by using information and communication technologies (3). Digital health involves wide range of stakeholders, including engineers, social scientists, public health practitioners, healthcare workers, and data analysts (4). Health systems are connected through digital health technology to improve computational techniques, use of smart devices, and communication channels to promote the health and well-being of patients and aid healthcare workers in managing patients’ health conditions (3). Digital health technologies comprise of technologies such as telemedicine, wearables, and virtual reality devices (5).

Several studies have been done to assess health information interchange, especially in developed countries where electronic records were established to some extent (6). The exchange of those records were also implemented, either entirely or partially (6). Moreover, precise patient matching is crucial to facilitate electronic health sharing.

This special issue entitled “Insights in Health Informatics: 2021” contains six articles to explore the latest technological refinements in Health Informatics. Researchers in this field will benefit from this article collection by providing them valuable information relevant to digital health. Most of the results, which are discussed in the subsequent paragraphs, focus on investigating digital health's impact on healthcare systems.

The effective integration of telemedicine into health and healthcare system has the ability to solve significant health crises such as accessibility to healthcare. Also, the evaluation of health professionals’ competence prior to telemedicine adoption is essential to improving global health systems. This issue has been investigated by Wubante et al. (10.3389/fdgth.2022.976566), where the impact of healthcare professionals’ understanding of telemedicine and its associated factors have been assessed at private hospitals in limited resource areas in Ethiopia. The authors adopted institution-based cross-sectional survey approach, where 423 healthcare workers from private hospitals participated between March and April 2021. The data were gathered using self-administered questionnaires. According to the authors, significant proportion of health professionals have a solid knowledge of telemedicine in these regions. However, more training for medical professionals is required to significantly improve the healthcare system and successfully deploy telemedicine, especially in area with limited resources.

Applying the science of health informatics to healthcare system has not received much attention until lately. The link between human health and climate change is still emerging. The improvement goal for health informatics needs to focus more on how climate change will affect human health. Methods for addressing this critical health issue with the help of health informatics are emerging recently. The research by Gray (10.3389/fdgth.2022.869721) provides a brief overview of how health informatics may lower the carbon footprint of healthcare and how it might incorporate novel data for understanding the effects of climate change on human health. Furthermore, health information and communication technology might be some of the driving factors through the production and disposal of medical technology and wearables, running data centres and telecommunications hubs, as well as the rising energy demands of data processing for health applications utilizing artificial intelligence and machine learning, are all potential sources of carbon dioxide emissions for the global digital health industry (7). According to Gray (10.3389/fdgth.2022.869721), health informatics can help to limit the specific effects of digital health by directing judgments for low-carbon health information technology infrastructure.

Furthermore, health informatics can demonstrate how to effectively use data-driven and digital health technology to reduce unnecessary over-working. Based on the Capurro et al. (8) investigation, algorithms for assessing massive datasets that are regularly obtained through clinics and patient records, for example, can be created as new digital screening technologies emerge to assist more precise diagnostic decisions and reduce the likelihood of overdiagnosis. Health informatics professionals can contribute to the response of climate health crisis by enhancing a better understanding of climate issues into the primary objectives, research institutes can host forums to foster creative thinking on the subject, and professional societies can develop ethical practice standards (Gray, 10.3389/fdgth.2022.869721).

The use of digital health (such as AI-based approach) to enhance healthcare system, which is becoming more popular recently, was studied by Milling et al. (10.3389/fdgth.2022.886615) and Williams et al. (10.3389/fdgth.2022.887015). A viable direction regarding national interoperability might be made possible by expanding data collection outside clinical workers and also by including public health practitioners to enhance interventions, boost surveillance, and fill awareness of health gaps for inspections. A review of recent developments in automatic speech-based disease diagnosis by Milling et al. (10.3389/fdgth.2022.886615), in particular, showed encouraging results for a number of cases. It is crucial to stress at this point that the future digital health in healthcare is not to take the place of medical professionals but rather to act as an additional examination tool that can assist them in detecting diseases and validating interventions more effectively and reliably. Moreover, Williams et al. (10.3389/fdgth.2022.887015) also suggested useful approach to enhance patient health information communication to efficiently achieve the target goals.

In healthcare settings, patient safety is crucial, including dental care. Dental practitioners gather up-to-date information of patients’ treatment history to prevent potential injury (9). This helps improve success during dental treatment (10). In their works, Shunning et al. (10.3389/fdgth.2022.838538) and Shunning et al. (10.3389/fdgth.2022.847080) investigated the effect of digital health on dental health and dental healthcare. In particular, the records from predoctoral dentistry student clinics were examined in Shunning et al. (10.3389/fdgth.2022.838538) to explore the backgrounds of consultations, patients’ history, as well as the responses, and the period required to get the information. According to their results, the most common requests for information were the laboratory diagnosis, existing medical problems, and prescription record.

Medical professionals routinely discuss the risks associated with dental procedures, prophylactic use of antibiotics, and contraindications, such as using local anaesthesia that contains vasoconstrictors. Shunning et al. (10.3389/fdgth.2022.838538) emphasized the significance of multidisciplinary collaboration among healthcare workers, such as dental and medical professionals, to deliver the best dental treatment as well as the requirement for suitable systems to improve information sharing and communication amongst health professionals to promote patient health condition. The recently expanded regional healthcare information exchange (HIE) is crucial approach that could help improve healthcare system; however, research on connecting dental clinicians with HIE is still being performed. The HIE approach was analysed in a study by Shunning et al. (10.3389/fdgth.2022.847080) to solve the inadequacies while maintaining the benefits of present techniques. The study modelled several features of current approaches to determine their strengths and weaknesses. Current technique models identify individuals, resources, organizational components, workflow, and places for development. For future development, it could inform software developers and other stakeholders about the system requirements, functionalities, and processes defined using HIE approach. The authors effectively simulated how dental clinicians access patient medical histories and developed a HIE strategy that addressed the shortcomings of the existing strategies while capitalizing on their advantages.

Finally, we suggest that there are many potential ways of which digital health technology adoption can be effectively implemented, which include the provision of appropriate knowledge and comprehension of the relevant experts’ guiding principles, learning, perception, and working environments (11).
